# Conducting Phase IV clinical studies: a moral imperative?

**DOI:** 10.3332/ecancer.2012.276

**Published:** 2012-10-26

**Authors:** TP Hill

**Affiliations:** Edward J. Bloustein School of Planning and Public Policy, Rutgers, The State University of New Jersey, USA and Neonatology; Division of the Robert Wood Johnson University Medical Center, New Brunswick, New Jersey, USA

## Abstract

The answer to this question lies in knowing the moral standing of Phase IV studies and whether we ought to conduct them. And to know this, in part, we need to compare them to studies in Phases I, II, and III and then determine where Phase IV studies stand in relation to Phase I–III studies scientifically and commercially.

## Introduction

The first thing to note about Phase IV studies is that they are more varied in design than the preceding clinical trials [1]. This variety is captured well by Suvan [2] who notes that it can include the research conducted after a drug or device has been approved for marketing. One form of this research is a non-interventional study (NIS) designed to assess a treatment’s safety, tolerability, and effectiveness in clinical practice. Another is the large simple trial (LST), combining aspects of the randomized clinical trial with those of an observational study. Yet another is post-marketing surveillance undertaken to determine safety, tolerability, and effectiveness in a particular population. Then there are retrospective case–control studies to assess what reasonably are thought to be rare side effects. Phase IV can also involve drug utilization studies (DUSS) which tell us how a drug is marketed and actually prescribed within a population. Finally, there is the registry or a prospective observational study of patients with a common condition or risk that can result in an accurate assessment of clinical practice, patient, and comparative outcomes.

What is of particular interest to an ethicist here is that the investment in Phase IV studies coincides with the point when the life-cycle of a treatment may be approaching the end of the patent period. Most, though not all, of the critical clinical questions have been resolved. As a result, from a commercial perspective, any interest in considering issues occurring after market approval may undergo a proportionate decline. This is particularly true when new drugs are under development specifically to replace older drugs. Nevertheless, Suvan concludes that monitoring spontaneously reported adverse events must continue for the duration of the drug’s usage. If so, what does this need say for the ethics of Phase IV studies?

## Moral standing of Phase IV trials

There is a scientific logic to the design of the first three phases which derives from their separate but complementary purposes. Having established the safety of a new drug or new combination of drugs in Phase I, we proceed to establish efficacy in Phase II. In Phase III, we compare the new drug or new combination with standard of care treatments to determine whether the new drug is as good as or better than standard treatment. Because the scope of these three phases remains of necessarily limited, there remain a number of critical questions to be answered even as the new drug or new combination is registered for commercial use. We don’t know whether there are additional patient populations or indications for which the new drug might be useful. We don’t know what the long-term effects of the new drug might be; nor do we know how the new drug will interact with any number of other drugs. Amidst some certainty about drug efficacy, there remains considerable uncertainty about drug effectiveness. And as we have learned from recent painful experience, the former is no substitute for the latter. Consider, for example, the questions raised over the use of aprotinin [3] to reduce loss of blood after cardiac surgery and the accompanying risk of harm from renal dysfunction and thrombosis. In the face of reasonable uncertainty, the use of aprotinin varied between universal, to reduce any risk of bleeding, and limited, to reduce a high risk of bleeding, after cardiac surgery. Despite the uncertainty which was in evidence even after drug approval, there were no reliable data with which to assess the risk of harms relative to the likelihood of benefits from aprotinin. Nevertheless, that did not seem to prevent “data-driven … efforts to expand the indications for the drug,” such as controlling inflammatory reaction following cardiac surgery. At the time, this was thought to call for increased dosage with the possibility of increased toxicity. If this was a reasonable conclusion, “until the safety of higher doses is fully explored in a prospective study, the expansion of indications for aprotinin may be premature”

Where then do we stand ethically with the successful completion of a Phase I–III series of a clinical trial? If the post-approval clinical history of aprotinin, which is by no means unusual, is anything to go by, there is a moral obligation, based on the twin principles of beneficence and non-maleficence, to determine the scientific warrant for expanding the indications of newly approved drugs. This is the role of Phase IV studies.

## Phase IV defined

According to the World Health Organization (WHO) [4], Phase IV studies are conducted after a drug has already been marketed. Their purpose is to monitor drug effectiveness in the general population, while collecting information about adverse effects associated with widespread use. Given this goal, Phase IV studies may be required by regulatory bodies or they may be conducted by the sponsoring pharmaceutical company to find new markets, to examine possible interactions with other drugs, or to determine outcomes with particular patient populations, such as pregnant women, the elderly, or those with rare tumours. Because they include endpoints like rare, long-term adverse effects, and involve much larger subject populations than those traditionally involved in Phase I–III studies, they are expected to last much longer than those earlier studies [5].

In light of these characteristics, can we say that Phase IV studies are of the same or a different kind from Phase I–III studies? Does it matter, ethically speaking, to make this determination? It does to the extent that there is a well-established critique for the earlier phases of clinical studies. Depending upon any significant differences between Phases I–III and Phase IV, that critique may have to be amended to account for the differences. They are certainly alike in their involvement of human subjects and in being subject to the requirements of scientific integrity. However, in one very significant regard, they are unalike in that they are more accurately defined as studies to demonstrate effectiveness rather than efficacy. Efficacy refers to “the extent to which a drug has the ability to bring about its intended effect under ideal circumstances, such as a randomized clinical trial” [6]. In contrast, effectiveness refers to “the extent to which a drug achieves its intended effect in the usual clinical setting”. Assuming the difference between the two is not merely semantic, what is the relationship between the two? According to Fritz and Cleland, the concepts of efficacy and effectiveness are not dichotomous but stages on a spectrum. “Many studies have characteristics of both. However, studies of treatment outcomes can generally be described as taking either an efficacy or an effectiveness approach”. The distinction is relevant when considering Phase IV studies precisely because they take place under conditions akin to standard care provided in the usual clinical setting. “Essential to Phase IV research is the focus on how drugs work in the real world” [7]. As a consequence, they involve a diversity of clinical settings together with a comparable diversity of patients and clinicians associated with these clinical settings.

## Internal and external validity

If we explore the implications of the distinction, we will see its ethical relevance. For example, it is clear that clinicians conducting Phase IV studies have other interests to serve besides those of their patients. This can lead, for example, to conflicts of interest [8] between the integrity of their research and the need to find evidence in support of the effectiveness of a drug already registered. In addition, the diversity of clinical settings can lead to uncontrolled conditions, such as clinicians not properly trained as investigators for the conduct of the research which in turn may compromise research integrity. We have also to consider the risks associated with bias in the selection of patients for the research. And there is the serious concern over confusing clinical research with clinical practice [9]. Yet another consideration is the danger that Phase IV studies, in the pursuit of expanded indications, become global exploitation, preying on patient populations in countries where the oversight of research involving human subjects is inadequate.

Since scientific integrity is a necessary but not sufficient condition for the ethical warrant of clinical research, the fact that Phase IV studies are known to use a greater variety of study design has ethical implications. Phase IV studies can also modify or dispense with methodological requirements, such as blinding, intended for internal validity. Nor it is unusual to see quasi research methodologies, such as a regression discontinuity study design being used instead of randomization [10]. And since Phase IV studies are conducted under “real world” conditions rather than the carefully controlled conditions of a Phase III study, treatment can be provided to participating patients on a more individualistic basis with the result that any scientific assessment of the outcomes may be problematic. This may explain Phase IV research’s emphasis on external validity as it focuses on the possible suitability of Phase I–III study findings for patient populations, clinical conditions, and clinical settings other than those associated with these earlier studies. But there are ethical implications for Phase IV studies specifically regarding external validity. One is the use of convenience sampling when accruing patients to a study. Another is the determination of criteria for the exclusion of patients. Associated with both of these is the use of specialized patient and clinician sampling, as well as specialized treatment conditions.

Turning to the internal validity of Phase IV studies, we should note the following serious challenges. The first is the need to design robust research and quasi research methods that are appropriate to the goal of expanding with scientific, and consequently ethical, warrant the indications for newly approved drugs. The second is the need to involve in the data analysis emerging techniques, such as biometrics. The third is that, given the critical relationship between internal and external validity, where internal validity is questionable the generalizability of the findings may be compromised. The relationship of scientific validity to ethics lies in the ethics caculus that the greater the scientific validity the less likelihood there is that patients participating in clinical research will be exposed unnecessarily to risk of harm.

## Institutional problems

In light of these observations, there is reason to agree with the assertion of Rajan [11] that a solid archetype in Phase IV studies is critical and will depend for its realization on what are described as unique levels of cooperation between clinical scientists and those responsible for marketing post-approval drugs. But here also Phase IV research faces serious difficulty, now at the institutional level, where it requires science and commerce to cooperate to a degree unprecedented for the pharmaceutical industry. As Rajan explains it, commercially Phase IV data are essential in generating post-approval safety information in order to extend the marketing of the drug. The same is true scientifically in order to confirm for physicians the therapeutic benefits of the drug for a wider range of indications in a larger population. The challenge lies in aligning market trends with patient needs, where the former can be variable and the latter are constant. The purpose of Phase IV is to function somewhere between these two asymmetrical but related forces so that they are mutually responsive. If successful, Phase IV research can be instrumental in meeting the needs of patients even as it responds to the priorities of payers, prescribers, and regulators. Is this possible?

Current literature suggests that serious challenges of both strategy and execution stand in the way of success. There is, for example, the tension within clinical development teams as they try to integrate pre-approval studies with marketing studies [12]. Paralleling this is the need for collaboration between investigators and the industry in order to align scientific goals with marketing objectives [13]. In part, this depends on industry policies regarding resources allocation for the entire Phase IV enterprise. To illustrate this point, consider that between 2008–2011 staffing for Phase IV studies increased by 85% compared with a staffing increase of 150% for Phase I and a tripling of staff for Phases II and III [14]. Because of the organic-like nature of phased clinical research, conducting Phase IV studies requires continuity in the form of planning them at least two years in advance of drug approval [15]. And since Phase IV studies involve larger patient populations, it is critical to have readily available to investigators patient registries. At present, about 30% of any one study’s timeline is dedicated to identifying patients suitable for Phase IV research purposes [16]. Related to this is the need, when contemplating a Phase IV study, to be prepared to meet the more demanding expectations of payers and regulators [17].

Many of these challenges can be traced to poorly aligned interests of the key players likely to be involved in Phase IV research. Expanded clinical indications and resulting market expansion understandably provoke concerns within the payer and regulatory establishments. Similarly, Phase IV studies seem to exacerbate both internal and external competitive pressures within the industry. A proposed robust Phase IV research agenda reveals habits of under-resourcing and the prohibitive duration of Phase studies, reliably longer than planned. It also exposes an understandable ambivalence in the attitude of clinical scientists who question whether scientific integrity is compatible with commercial objectives.

Nevertheless, overarching these misaligned interests, there is an underlying agreement among those key to the success of the Phase IV enterprise that it is and will remain indispensable. The research is necessary to confirm the legitimacy, post-approval, of expanding clinical indications, thereby meeting the needs of a larger patient population. This, in turn, is a necessary condition for any legitimate expansion of the marketing, post-approval, of the drug. Furthermore, it is hard to see how the expectations of both payers and regulators are to be met without a sustained Phase IV program.

If nothing else, this confirms the need, alluded to earlier, for Phase IV studies in all their varied designs and with their varied goals. Meeting the need means addressing the already widely acknowledged scientific and institutional challenges. No doubt serious, they are nonetheless certainly surmountable, evidence for which can be found in the fact that they are being conducted, even as we speak.

## Contractarianism

If then, as this article contends, there is a moral obligation to conduct Phase IV research in order to address the clinical consequences, potentially positive and negative for an as yet unproven patient population, what can ethics provide to help those involved to meet their obligation? One promising strategy is a social contract paradigm, known as contractarianism [18]. Its purpose is to secure the common good, ethically interpreted, by reconciling conflicting or misaligned values, also interpreted ethically. Contractarianism, as envisioned here, is premised on three considerations. The first is an initial bargaining position which reflects basic interests as perceived by each of the key players involved. The second is a characterization of each of the key players as rational and motivated to come to a working agreement. The third consideration is a set of conditions already in place and by means of which the necessary alignment of interests is both possible for and beneficial to those involved. In other words, the paradigm, applied to the Phase IV enterprise, presumes that the starting position of investigators, regulators, payers, and policy makers is demonstrably reasonably grounded. It also presumes that current conditions and trends affecting Phase IV research indicate that alignment of interests is both possible and would be beneficial. Given these conditions, in particular the acknowledgment of an underlying rationality common to all the key players, the paradigm dictates their coming together to align their interests in such a way as to secure the common good in this case in the form of an appropriate model for use in the Phase IV research enterprise.

The moral substance of this argument comes from the understanding that ethics can be derived from rationality. This is the position of Gauthier who contends that while human beings do not enjoy a natural harmony of interests, cooperation among human beings promises them considerable gains. However, given the preponderance of individual self-interest as the condition natural to human beings, being able to impose a constraint, morally justified, on the individual’s preferred self-interest is essential to avoid what is known in game theory as the prisoner’s dilemma or the belief that from the individual’s perspective the best results come from cheating on an agreement while the other individual’s party to an agreement observe its terms. Being disposed to act on the basis of such a belief would, in effect, leads to a general expectation of being cheated. And if this is the prevailing context of every agreement or contract, any agreement or contract would be compromised from the start. The antidote is for one to see as rational and therefore moral acting according to the terms of the contract when the other parties are also so inclined.

This prompts consideration of what Gauthier calls the “compliance problem,” referring to what persuades parties to an agreement that compliance with the terms of the agreement is rational. According to Gauthier, there are two things to consider here. One is the initial situation of each of the parties and the other is the conduct of the situation to which the parties have contracted themselves, in other words, the bargain. Critical to the conduct of the bargain are the ethical norms that would, in the view of the bargainers, result in their greatest advantage. Gauthier envisages this outcome as consisting in what he calls the “minimax relative concession” or those concessions one bargainer is willing to make in response to the concessions of the other bargainers. Presuming the concessions of any one bargainer are reasonable in relation to the concessions of the other bargainers and expecting to maximize one’s advantage while securing an agreement and compliance following the agreement, it is the bargainers come to see rational to agree to the contract. If it is the presumption that the concessions of any one bargainer are reasonable in relation to the concessions of the other bargainers, and if the expectation is to maximize one’s advantage while securing an agreement and compliance following the agreement, then the bargainers can be assured that it is rational to agree to the contract. Given these conditions, the most reasonable, and therefore ethical, result for Gauthier would be one in which the greatest concessions made by each bargainer become, in the process of bargaining, lessened.

As critical as the conduct of the bargain is for Gauthier, so also is the initial situation of the bargainers. That is, if the agreement is to be rational, the bargainers they will have had to find themselves there freely. An indispensable condition for Gauthier, it reflects an understanding on the part of each bargainer that his or her advantage cannot come from disadvantaging the other bargainers.

As a strategy, Gauthier’s version of contractarianism has to assume that in the absence of a contract, the prevailing circumstances in which each of the parties find themselves is unworkable. In acknowledging this, they find sufficient reason to pursue reciprocated concessions designed sufficiently to surmount the original inability to collaborate in the absence of a contract and thus, but now with an agreement, reasonably ensure compliance with the terms of the contract.

In ethics, no one is obliged to fulfil the impossible. It may be argued that the problems, both scientific and institutional, discussed here render a sustained Phase IV agenda extremely difficult if not impossible. Of the two sets of problems, the scientific problems are the most easily addressed. And while the institutional problems pose serious difficulties as we try to reconcile the quite disparate objectives of patients’ well being, clinical research, regulation, insurance, and health policy converging in the Phase IV enterprise, the strategy stipulated in Gauthier’s contractarianism provides a reasonable and practical approach that would put a universally acceptable research model within reach. In which case, if we know how it can be done, and done for the moral good embodied in the concept of the Phase IV enterprise, then we ought to conduct, as a matter of course, Phase IV research.
